# Application of New Drugs in Chronic Lymphocytic Leukemia

**DOI:** 10.4084/MJHID.2010.011

**Published:** 2010-05-10

**Authors:** Tadeusz Robak

**Affiliations:** Department of Hematology, Medical University of Lodz and Copernicus Memorial Hospital, Lodz, Poland

## Abstract

Over the last few years, several new agents have been under evaluation in preclinical studies as well as in early clinical trials, and have shown promise in treating CLL. These treatments include new monoclonal antibodies (mAbs), immunomodulating agents, novel purine nucleoside analogs, Bcl-2 inhibitors and other agents. The most promising are a new mAbs targeted CD20 molecule or CD23, anti-CD40 mAbs and anti-CD37 antibody. Oblimersen, flavopiridol, and lenalidomide are also being evaluated both in pre-clinical studies and in early clinical trials. However, available therapies are only partially efficient and there is an obvious need to develop better strategies and new, more specific and active drugs.

## Introduction:

For many years, alkylating agents and purine nucleoside analogs (PNA) have been considered the drugs of choice for treatment of CLL.^1^,^2^ Combination therapies with PNA and cyclophosphamide are more active than monotherapy in terms of overall response rate (OR), complete response (CR) and progression free survival (PFS).^3^–^6^ Recent reports suggest that the administration of monoclonal antibodies (mAbs) can significantly improve the course of CLL.^7^ At present, there are two antibodies with great clinical value for patients with CLL. The first is rituximab (Rituxan, Mabthera) a chimeric anti-CD20 mAb that targets CD20 antigen.^8^ The CD20 antigen is expressed on almost all B-cells in patients with CLL but the intensity of expression appears to be lower than in patients with non Hodgkin lymphoma (NHL). Rituximab in conventional doses of 375 mg/m^2^ weekly for 4 doses has rather low activity in CLL. However, some studies suggest that higher doses are more effective than standard doses, used routinely in other lymphoid malignancies.^9^ The second approved mAb is alemtuzumab (Campath-1H), a humanized therapeutic mAb that recognizes the CD52 antigen expressed on normal and neoplastic lymphoid cells.^10^ This mAb is active in previously treated patients with CLL refractory to PNA. Alemtuzumab was also investigated in previously untreated patients with this leukemia. The results of a prospective randomized phase III study (CAM 307 trial) comparing high-dose chlorambucil with alemtuzumab in the first-line treatment of progressive CLL were recently published.^11^ The OR rate, CR rate, and PFS time were superior for alemtuzumab. Alemtuzumab is an effective drug in CLL patients with poor risk cytogenetics, such as deletions in 17p. However, alemtuzumab is ineffective in patients with bulky nodal disease (>5 cm). In previously untreated patients with CLL, an OR rate of more than 80% can be achieved.^4^,^5^

In randomized trials the combination of rituximab with fludarabine and cyclophosphamide (R-FC) demonstrated higher OR rate and CR rate, and longer PFS time than F C in previously untreated and relapsed/refractory CLL^12^,^13^ Recently several new agents have been explored and have shown promise in CLL.^14^,^15^ Novel therapies are being evaluated both in pre-clinical studies and in early clinical trials. These treatments include new monoclonal antibodies, agents targeting the antiapoptotic bcl-2 family of proteins, receptors involved in mediating survival signals from the microenvironment, antisense oligonucleotides and other agents.

## Novel Monoclonal Antibodies:

Over the last few years, several new mAbs and have been investigated in clinical trials in patients with CLL (**Table 1 and Table 2**).^16^,^22^

Ofatumumab (HuMax-CD20; Arzerra, GlaxoSmithKline/Genmab), is a second-generation, fully human, anti-CD20, IgG1 mAb.^16^–^18^ Ofatumumab recognizes a different CD20 epitope to rituximab. Compared with rituximab, ofatumumab has similar antibody-dependent cellular cytotoxicity (ADCC) but stronger complement-dependent cytotoxicity (CDC) and does not induce cell death by apoptosis.^17^ Ofatumumab has been investigated in phase I/II study in 33 CLL patients with refractory or relapsed diseases.^16^ In patients treated with doses of 2000 mg, the objective response rate was 50%. Subsequently, an international pivotal trial of ofatumumab in patients with CLL refractory to both fludarabine and alemtuzumab, or refractory to fludarabine with bulky lymphadenopathy has been undertaken (Hx-CD20-406 phase III study).^18^ The data from this study indicate that the activity and safety profile of ofatumumab appeared to be consistent with the prior phase I/II study. The overall reponse rate (ORR) was 51% for the patients with CLL refractory to fludarabine and alemtuzumab and 44% for the patients refractory to fludarabine with bulky lymphadenopathy. Median time to next CLL therapy was 9 months and 8 months, respectively. There were no unexpected toxicities. These preliminary results demonstrate promising efficacy of ofatumumab monotherapy in a heavily-pretreated patients with fludarabine-refractory CLL. Ofatumumab potentially represents an active treatment option with clinical benefit for patients with very poor prognosis who have exhausted standard treatment options.

GA-101 (RO5072759, Hoffman La Roche and Genentech) is a novel, third-generation, type II, anti-CD20, fully humanized, IgG1 mAb, different from rituximab.^19^–^21^ GA-101 binds with high affinity to the CD20 epitope and, as a result, induction of ADCC is 5–100 times greater than with rituximab. It also exhibits superior caspase–independent apoptosis induction than rituximab. However, CDC activity is low.^21^ In the phase I/IIa study GA-101 was administered as a single agent to 24 patients with CD20^+^ malignant disease, who were heavily pretreated, virtually all with prior rituximab and for whom no therapy of higher priority was available.^20^ Patients were treated with GA-101 at doses from 50 mg to 2000 mg. The antibody has shown a similar safety profile to rituximab and has demonstrated promising efficacy in this difficult-to-treat patient population.

Lumiliximab is a genetically primatized, macaque human chimeric anti-CD23 IgG1κ mAb investigated for the treatment of relapsed CLL.^22^–^25^ It induces ADCC and CDC, and enhances apoptosis when combined with current or emerging CLL therapies including chlorambucil, fludarabine, alemtuzumab and rituximab.^22^ Byrd et al report the results of a phase 1–2 study, testing the R-FC regimen combined with lumiliximab in 31 patients with refractory/relapsed CLL.^25^ The patients had a median of 2 prior therapies (range 1–10) and 61% of them were treated with fludarabine. The OR rate was 65%, including 52% CRs. The median PFS was 30.4 m (range 9.8–47.7m). In terms of CR rate, R-FC+lumiliximab in this study compared favorably with the activity of R-FC in a similar patient population (25% CRs) previously reported by Wierda et al.^26^ However, the PFS was similar in both trials: 29m and 28m, respectively. Importantly, the addition of lumiliximab to R-FC treatment did not increase its toxicity. Further randomized studies are ongoing to confirm that the use of lumiliximab in combination with R-FC is a more effective treatment regimen for the eradication of CLL.

TRU-016 is an intravenously administered anti-CD37 IgG fusion protein for the potential treatment of B-cell malignancies, including CLL and non-Hodgkin's lymphoma (NHL).^27^,^28^ TRU-016 was created by humanizing SMIP-016, a mouse/human chimeric protein with preclinical antitumor activity against lymphoid malignancies. TRU-016 was active in mouse human B-cell tumor xenograft NHL models. In addition, TRU-016 demonstrated synergistic or additive activity in NHL cells in combination with rituximab, rapamycin, doxorubicin and bendamustine. In a phase I clinical trial in refractory or relapsed patients with CLL or small lymphocytic lymphoma, TRU-016 was well tolerated with clinical benefit and a reduced absolute lymphocyte count observed in all cohorts above the 0.1-mg/kg dose.^28^

In addition, several other mAbs directed against lymphoid cells have been recently developed and investigated in preclinical studies and clinical trials (Table 1).^14^ These treatments include epratuzumab, galiximab and anti-CD40 mAbs.

## Novel Purine Nucleoside Analogs:

Recently three novel PNAs: clofarabine (CAFdA), nelarabine (ara-G) and forodesine (immucillin H, BCX-1777), have been synthesized and introduced into clinical trials.^29^,^30^

**Figure 1.**
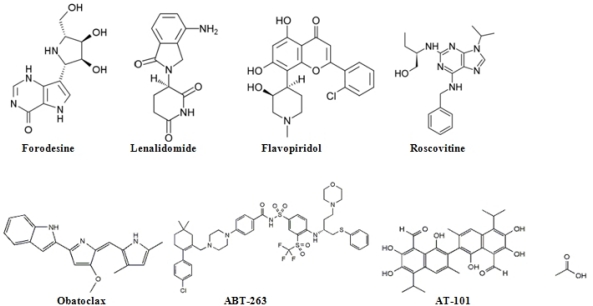
Chemical structures of new drugs, potentially useful for chronic lymphocytic leukemia.

Forodesine (Immucillin H) is a type of immucillin: a 9-deazanucleoside analogue which acts as a purine nucleoside phosphorylase (PNP) inhibitor.^29^ Immucillins have a structure based on ”nitrogen in the ring” D-ribofuranosyl C-glycosidic analogues of natural nucleosides. Forodesine is the most potent member of this group (**Figure 1**).^29^ Recently, preliminary studies have been initiated into the efficacy of forodesine in the treatment of patients with advanced and refractory CLL.

## Immunomodulating Agents:

Immunomodulating agents are a new class of drugs that change expression of various cytokines and costimulate immune effector cells.^31^–^35^ Thalidomide and its derivative lenalidomide (Revlimid; Cellgene) are immunomodulating agents with possible antiangiogenic properties which modulate cytokine activity in the tumor microenvironment. The exact mechanism of action of this drug remains unknown although antiangiogenic and immunomodulatory effects through cytokine modulation in the tumor microenvironment have been reported. Lenalidomide is a less toxic analog of thalidomide, and *in vitro* has more potent activity than the parent compound (Figure 1).^31^ This agent has been investigated in patients with relapsed/refractory and previously untreated CLL. Chanan-Khan et al.^32^ reported the anti-leukemic effects of lenalidomide in 45 CLL patients with relapsed or refractory disease. The drug was administered orally at a dose of 25 mg once a day for 21 days on a 28-day schedule. Major responses were observed in 21 patients (41%), with 4 CR (9%), and 17 (38%) achieving a PR in the intent-to treat analysis. The most common non-hematologic adverse events were fatigue (83%) and flare reaction (58%). Ferrajoli et al.^35^ presented the results of a phase II study in which lenalidomide was started with lower doses of 10 mg per day by continuous daily dosing, with dose escalation up to 25 mg, based on patient tolerability and response. Three out of 44 patients (7%) achieved a CR and OR rate was 32%. Thirteen patients (30%) developed tumor flare reaction.

Recently, Chen et al.^34^ have reported preliminary results from a phase II study of lenalidomide used as a single-agent in previously untreated, symptomatic CLL. The starting dose for lenalidomide was initially 10mg po daily with weekly 5mg dose escalations to the target dose of 25mg daily x 21 days every 28 day cycle. All 17 patients, evaluable for response, have achieved PR (65%) or stable disease (35%). Responses were reached at a median of 4 cycles (range 2–15). Preliminary results from this phase II study suggest that lenalidomide has a significant activity and acceptable toxicity in previously untreated CLL patients. However, a conservative low-dose, continuous dosing regimen and careful safety monitoring are suggested to obtain a safer and more effective use of this drug.

Ferrajoli et al.^35^ evaluated the efficacy and tolerability of lenalidomide as initial therapy of older patients with CLL. All patients received the drug at the dose of 5 mg daily for the first 56 days and then the dose could be increased up by 5 mg every 28 days to reach a maximum dose of 25mg daily. Nineteen (54%) patients achieved a PR and 14 patients (40%) had stable disease. These results indicate that lenalidomide given as continuous therapy at a start dose of 5 mg followed by slow dose escalation is safe and well-tolerated in initial therapy of elderly patients with CLL.

Bcl-2 Family Inhibitors: Oblimersen (Genasense, Bcl-2 antisense, G3139) is a synthetic, 18-base, single strand phosphorothioate DNA oligonucleotide designed to down-regulate *Bcl-2* mRNA expression.^36^,^37^ The agent recognizes the first six codons of Bcl-2, forming a DNA/RNA complex that inhibits translation of the protein. Oblimersen consequently reduces levels of Bcl-2 expression, reduces cell viability, increases activity of pro-apoptotic mechanisms, reduces tumor size and enhances anticancer drug activity. The addition of oblimersen to chemotherapy with fludarabine and cyclophosphamide (FC) produced a significant increase in the number of durable remissions in patients with relapsed or refractory CLL.^37^ In addition, the updated analysis showed that in relapsed/refractory CLL, oblimersen combined with FC offers patients who achieve CR or PR, as well as those who have fludarabine-sensitive disease, a significant survival benefit.^37^ This agent should be further evaluated in previously untreated patients. Therapeutic modulation of the Bcl-2 pathway may represent a new treatment option in CLL.

Obatoclax (GX15-070) is a hydrophobic molecule, developed as a Bcl-2 family antagonist (**Figure 1**).^38^,^39^ This agent inhibits several anti-apoptotic Bcl-2 family proteins including Bcl-X_L_, Bcl-2, Bcl-w, BCL-B, A-1 and Mcl-1. Moreover, obatoclax can promote the release of cytochrome C from mitochondria isolated from leukemia cells. Apoptosis induced by this agent was preceded by the release of Bak from Mcl-1, liberation of Bim from both Bcl-2 and Mcl-1 and the formation of an active Bak/Bax complex.^38^ O’Brien et al.^40^ reported the results of a phase I trial of obatoclax in CLL patients. The drug was administered to patients with advanced CLL both as a 1-hr infusion at doses ranging from 3.5 to 14 mg/m^2^, and as a 3-hr infusion at doses between 20 to 40 mg/m^2^ every 3 weeks. One (4%) of 26 patients achieved a PR. Patients with anemia (3/11) or thrombocytopenia (4/14) experienced improvements in hemoglobin and platelet counts. Circulating lymphocyte counts were reduced in 18/26 patients with a median reduction of 24%. The most frequent adverse event was somnolence and euphoria occurring during the infusion or shortly afterwards.

ABT-263 is another small molecule that binds with high affinity to several anti-apoptotic Bcl-2 family proteins including Bcl-X_L_, Bcl-2 and Bcl-w but not Mcl-1 or A1(**Figure 1**).^42^,^42^ Oral bioavailability of ABT-263 is 20% to 50% depending on the formulation. The phase I/IIa trial was performed in patients with refractory or relapsed lymphoid malignancies.^42^ ABT-263 was administered at doses of 10, 20, 40, 80 and 160 mg to 17 subjects who took part in the study. Two patients with bulky CLL/SLL in the 40 and 160 mg cohorts had 95% and 64% tumor reductions

AT-101 ((-)Gossypol, Tw-37) is another orally-available small molecule that mimics the BH3 domain of cellular Bcl-2 and interferes with the function of prosurvival Bcl-2 proteins (Figure 1).^43^ It is a derivative of a natural product - gossypol - capable of binding to Bcl-2, Bcl-X_L_ and Mcl-1, attenuating their anti-apoptotic influence. AT-101 was investigated together with rituximab in a phase II study in refractory CLL patients by Castro et al.^44^ AT-101 was administered at a dose of 30 mg/d for 21 or 28 days during each of 28- day cycles. Rituximab was administered at 375 mg/m^2^ in 12 doses. The OR rate was 38%, grade 1–2 gastrointestinal toxicity occurred in 11 of 12 patients.

## Protein Kinase Inhibitors:

Flavopiridol, (Alvocidib, HMR-1275, MDL-107826A, NSC-649890) is a synthetic derivative of the flavonoid rohitukine (**Figure 1**).^45^ Flavopiridol was originally described as an inhibitor of cyclin-dependent kinase and other protein kinases because of its interaction with adenosine triphosphate binding sites. Recently presented up-dated results confirm that flavopiridol induces durable responses in heavily pretreated, relapsed CLL patients with bulky lymphadenopathy (>5cm) and poor-risk cytogenetic features.^45^

Roscovitine is a purine analog that competes with ATP for a binding site on cyclin-dependent kinases (CDKs) (**Figure 1**).^46^ This small molecule inhibits CDK-induced apoptosis in isolated CLL cells by caspase activation and modulation of Bcl-2 family proteins.^46^ Roscovitine and its pure R-enantiomer CYC202 (R-Roscovitine, Seliciclib) are independent of p53 activation or defects in p-53 dependent pathways. CYC2002 was shown to be highly effective in a panel of 19 human tumor cell lines and a human tumor xenograft model.^46^ In the CLL cell culture, CYC 202 displays reduced and delayed apoptosis. Moreover, this agent causes a significant down-regulation of genes involved in the transcription, translation, survival and DNA repair of CLL cells.^46^ CYC202 is a promising candidate drug for clinical tests alone or in combination with other agents in relapsed CLL.

## Conclusions:

Currently available therapies are only partially efficient in CLL, exposing an obvious need to develop new, more specific and active drugs. Recently, several new drugs have been developed and are being evaluated in clinical trials (**Table 2**). Further studies should elucidate the role of these new agents and their combinations in the management of CLL.

## Figures and Tables

**Table 1.** Newer monoclonal antibodies potentially useful for chronic lymphocytic leukemia

**MoAb**	**Antigen**	**Antibody characteristics**	**Clinical development**
**Ofatumumab (HuMax -CD20)**	CD20	Human IgG_1-κ_	PhaseI/II in CLL and NHL
**Veltuzumab (hA20, IMMU-106)**	CD20	Human IgG_1-κ_	PhaseI/II in NHL
**GA-101**	CD20	Humanized IgG1	PhaseI/II in NHL and CLL
**Lumiliximab (IDEC-152)**	CD23	Chimeric macaque/human	Phase III in CLL
**Epratuzumab (hLL2)**	CD22	Humanized IgG_1-κ_	PhaseI/II in NHL
**Galiximab (IDEC-114)**	CD80	Chimeric human/macaque IgG1	Preclinical studies
**HCD122(CHIR-12.12)**	CD40	Human IgG1	Phase I in CLL
**SGN-40**	CD40	Humanized IgG1	Preclinical studies
**TRU-016 (SMIP, CytoxB37G)**	CD37	Humanized fusion protein derived from anti-CD37 antibody	Phase I in CLL

Abbreviations: CLL –chronic lymphocytic leukemia; NHL – non-Hodgkin lymphoma

**Table 2.** Clinical trials of new agents in chronic lymphocytic leukemia

**Drug**	**Phase Of the study**	**No**	**Patients characteristics**	**Response**		**Median response duration**	**References**
				OR	CR		

**Ofatumumab**	I/II	33	Relapsed/refractory	44%	0%	PFS -106d	Coiffier et al.(6)

**Ofatumumab**	III	138 FA ref-59 BF ref-79	Relapsed/refractory	FA ref-58% BFref - 47%	BFref-1pt	FA ref-5.7m BFref-5.9m	Wierda et al. (8)

**GA-101**	I	13	Relapsed/refractory	62%	0%	NR	Salles et al (10)
**Lumiliximab**	I	46	Relapsed/refractory	28%^*^	0%	NR	Byrd et al.(14)
**Oblimersen**	I/II	40	Relapsed/refractory	8%	0%	NR	O’Brien et al.(18)
**Flavopiridol**	II	42	Relapsed/refractory	45%	0%	PFS 13 m	Byrd et al.(23)
**Lenalidomide**	II	45	Relapsed/refractory	47%	9%	NR	Chanan-Khan et al.(21)
**Lenalidomide**	II	44	Relapsed/refractory	32%	7%	2pts progressed	Ferrajoli et al.(22)

* Decrease in absolute lymphocyte count < 50%; NR – not reported; d-day; PFS – progression free survival; p.o. – orally; i.v. –intravenously; OR- overall response; CR- complete response, d –day; w – week; m – months; FA-ref - fludarabine- and alemtuzumab-refractory ; BF-ref - fludarabine-refractory CLL with bulky lymphadenopathy
